# Hepatitis B immunization status and risk factors of people aged 1 to 69 in Huangpu District, Shanghai, China

**DOI:** 10.3389/fpubh.2023.1302183

**Published:** 2023-12-14

**Authors:** Yijun Wang, Min Shu, Jun Chen, Fujie Shen, Hong Ren, Yongfu Yu

**Affiliations:** ^1^Department of Viral Hepatitis Prevention and Molecular Biology Laboratory, Huangpu District Center for Disease Control and Prevention, Shanghai, China; ^2^Department of Viral Hepatitis Prevention, Shanghai Municipal Center for Disease Control and Prevention, Shanghai, China; ^3^Department of Biostatistics, Key Laboratory of Public Health Safety of Ministry of Education, Key Laboratory for Health Technology Assessment, National Commission of Health, School of Public Health, Fudan University, Shanghai, China

**Keywords:** hepatitis B, immunization, HBsAb, HBcAb, HBsAg, multivariate analysis

## Abstract

**Background:**

China has long been with high Hepatitis B Virus(HBV) prevalence in the world. The HBV prevalence of people aged 1–59 decreased to less than 8% in 2006, and by 2020, HBsAg positive rate of children aged <5 decreased to less than <1% which was due to the free three-dose hepatitis B(HepB) immunization for newborns nationwide since 2002. Huangpu district was selected as one of the pilot areas for free Hep B vaccination in newborns since 1986, which formed an early protection in the population from mother-to-child transmission. However, the existed HBV infected people were still needed to be discovered, evaluated whether to receive antiviral therapies and intervened with health education in order to reduce the incidence of viral hepatitis related hepatocellular carcinoma (HCC) and also reach the goal to eliminate public health hazards of viral hepatitis by 2030.

**Objective:**

To know HepB immunization status among people aged 1 to 69 in Huangpu district of Shanghai, and find out risk factors changes of HBV infection.

**Methods:**

Cross-sectional study was applied to analyze the HepB immunization status and related risk factors by carrying out survey among 706 participants aged 1 to 69 years old. Blood samples were collected for detection of serological HBV markers including hepatitis B surface antigen(HBsAg), hepatitis B surface antibody(HBsAb) and hepatitis B core antibody(HBcAb). Participants with HBsAg positive were required to complete additional examinations such as alanine aminotransferase(ALT), aspartate aminotransferase(AST), total bilirubin, albumin, globulin, liver fibroscan and liver ultrasound.

**Results:**

For participants aged 1 to 14, the positive rate of HBsAg, HBsAb and HBcAb was 0.00, 50.00 and 30.46%, respectively. The HBsAb positive rate reached a peak of 90.91% at 2 years old, and then showed a significant downward trend (χ^2^ = 55.612, *p* < 0.001). All the participants have completed three-dose Hep B vaccination, however for the second dose, those who vaccinated 30 days later than the appointed time(aged one month) got higher HBcAb prevalence than those who vaccinated on time(χ^2^ = 5.87, *p* = 0.015). Two mothers were found HBsAg positive, but there was no significant difference in children’s HBcAb positive rates regardless of the mothers’ HBsAg results. For participants aged 15 to 69, the positive rate of HBsAg, HBsAb and HBcAb was 4.21, 44.25 and 49.23%, respectively. Multivariate analysis for HBcAb positive among people aged 15 to 69 showed that age(50–69) and HBsAb positive were the risk factors for HBcAb positive(*p* < 0.05). Higher education was the protective factor for HBcAb positive(p < 0.05). After the screening for HBsAg, 22 participants were tested HBsAg positive and required additional examinations, and a total of 12 completed all the examinations. One participant was recognized as active HBV infection without antivirus treatment. Among the 12 participants, 2 have received antiviral treatment before and 4 had a history of HBV infection in family members.

**Conclusion:**

In this study, HBsAg positive rate of those who aged 1 to 14 was 0.00%, which indicated that the HepB immunization has achieved a lot in protecting children from being infected. However, failing to get timely Hep B vaccination could be an influencing factor for HBcAb positive in children. As a result, additional tests for HBV DNA could be done to specify an HBV infection and more attention should be paid to the timeliness of Hep B vaccination in the next step. The HBcAb positive rate of people aged 1 to 69 was relatively higher than that of other provinces. Despite of the limited participants with full examinations, we should still put emphasis on HBV treatment and the possibility of transmission within families.

## 1 Introduction

Viral hepatitis B(HepB) is an infectious disease characterized by liver damage caused by hepatitis B virus(HBV) infection. HBV could cause acute infection and progress into chronic HBV infection in the absence of standardized antiviral treatment. Furthermore, chronic HBV infection can progressively lead to liver cirrhosis, which is known as an independent risk factor for hepatocellular carcinoma (HCC) (1). It was indicated that approximately 15–25% of chronic HBV infection population will finally die from cirrhosis or HCC in World Health Organization(WHO)'s Western Pacific Region(WPR) which has the highest rates of HBV infection in the world (2). In 2019, there were 296 million cases of chronic hepatitis B worldwide and approximately 820,000 deaths from HBV infection related diseases (3). There are still about 93 million people living with chronic HBV infection in China, and about 300 thousand people die each year from chronic HBV infection related diseases, such as cirrhosis and HCC (4).

China, with a higher hepatitis B surface antigen(HBsAg) positive rate than 8% in 1992, was then considered to be a country with high hepatitis B prevalence in the world according to the standards of World Health Organization(WHO). Afterwards, China began to promote HepB vaccination for newborns to cut off the mother-to-child transmission, which is responsible for approximately half of the HBV transmission (5). Later in 2002, free three-dose HBV vaccination was included into the China’s national immunization program for newborn babies. When it comes to 2010, the combination of HBV vaccine and hepatitis B immunoglobulin(HBIG) was offered for free to the neonates of HBsAg positive pregnant women. In the past 30 years, China has made remarkable achievements in HBV infection control, and the positve rate of HBsAg has decreased significantly. According to the China’s national sero-epidemiological survey of HBV infection (6, 7), the positive rate of HBsAg in the population aged 1–59 in 1992 was 9.75%. The positive rate of HBsAg in people aged 1–59 in 2006 was 7.18%, and the positive rate of HBsAg in people aged 1–4, 5–14 and 15–29 in 2014 was 0.32, 0.94 and 4.38%, respectively. WHO’s Western Pacific region has set one of the goals to achieve by 2017 which including reducing children’s HBsAg positive rate to <1% (2). China has reached the goal for children in advance (8), and continuously made efforts to decrease the HBsAg positive rate in the whole population, which was estimated at 6.1% in 2016 (9).

Huangpu district, as one of the pilot areas within Shanghai, China, has started HBV vaccination in newborns since 1986, which was quite earlier than nationwide vaccination. The serum hepatitis B surface antibody(HBsAb) levels were continuously monitored among HBV vaccinated population. Our previous study showed that the serum HBsAb still existed in about 88% children aged 9–12 years after three-dose HBV vaccination (10). Due to the long-term monitoring of HBV prevalence status and solid foundation of HBV prevention works, the overall HBsAg positive rate in Huangpu district was pretty lower than other districts in China. However, prevention of HBV infection still face many challenges. For example, frequent population movement would make it more difficult to do interventions. So regular sero-epidemiological study was carried out in Huangpu district to make clear local HBV prevalence status every few years. On the basis of the results, interventions would be discussed and altered to improve HBV prevention effectively. This study was carried out in 2020–2021, following the instructions made by China’s National Center for Disease Control and Prevention(China’s National CDC), mainly to monitor HBV prevalence and find local risk factor changes in HBV infection.

## 2 Materials and methods

### 2.1 Participants and recruitment

According to the requirements of the 2020 National Viral Hepatitis Immunization Effect Evaluation Survey, a multi-stage stratified random sampling method was used to recruit participants from the permanent population aged 1–69 in two monitoring neighborhood committees, which were named as Laoximen committee and Nandong committee in Huangpu District, Shanghai. Stratified random sampling was done separately in the two committees. Firstly, each resident was given an unique code according to building and room numbers. Secondly, the residents were allocated into four age groups, which were 1–4, 5–14, 15–29 and 30–69 years old. Thirdly, random sampling was done within each age group at an enlarged sample size of 43, 61, 87 and 215, which made 406 in each community and 812 in total, considering 15% rejection to the survey. Finally, survey should be carried out following the name list of residents until a required sample size of 74, 106, 152 and 374 in the four age groups was completed. The overall respondent rate in Huangpu district was 87.92%(706/803), with a respondent rate of 90.74%(353/389) for Laoximen committee and 87.81%(353/402) for Nandong committee.

### 2.2 Research content

All participants signed informed consent forms. The questionnaire was filled in through face-to-face investigation for the respondents. According to the instructions of China’s national CDC, the “2020 National Chronic Viral Hepatitis Epidemiological Survey Case Questionnaire (Applicable Form for Children 1–14 Years Old)” was used for people aged 1–14 years old, mainly collecting basic personal information, immunization history, HBsAg results of mothers and other information. People aged 15–69 years old used the “2020 National Chronic Viral Hepatitis Epidemiological Survey Case Questionnaire (Applicable Form for People aged 15–69)” to mainly collect basic personal information, history of viral hepatitis, related knowledge, etc. For those who were tested positive for HBsAg, addtional examinations like alanine aminotransferase(ALT), aspartate aminotransferase(AST), total bilirubin, albumin, globulin, liver fibroscan and liver ultrasound should be completed. Participants aged 1–14 years old were set to collect 3 mL of venous blood using negative pressure blood collection tubes, and 5 mL of venous blood was collected from subjects aged 15–69 and sent to the Shanghai Center for Disease Control and Prevention for serological HBsAg, HBsAb and hepatitis B core antibody(HBcAb) tests.

### 2.3 HBV serum marker testing

The detection reagents issued by the Institute of Viral Disease Prevention and Control of the China’s National Center for Disease Control and Prevention were used to carry out the initial screening of three serological indexes: HBsAg, HBsAb and HBcAb, and the detection method was enzyme-linked immunosorbent assay. The kits used for preliminary screening and review are produced by Beijing Wantai Biopharmaceutical Co., Ltd. and Abbott Laboratories of the United States, respectively.

### 2.4 Data analysis

The questionnaire data was uniformly entered into the “Epidemiological dynamic data collection platform” of the China’s National Center for Disease Control and Prevention. The data was exported to Excel 2007 for data cleaning, and statistical analysis such as chi-square test, trend test, logistic regression and so on was carried out by SPSS16.0.

### 2.5 Ethical review

This survey was reviewed and approved by the Ethics Committee of the Chinese National Center for Disease Control and Prevention (No.201811) prior to implementation.

## 3 Results

### 3.1 General information of the participants

**Figure 1:**
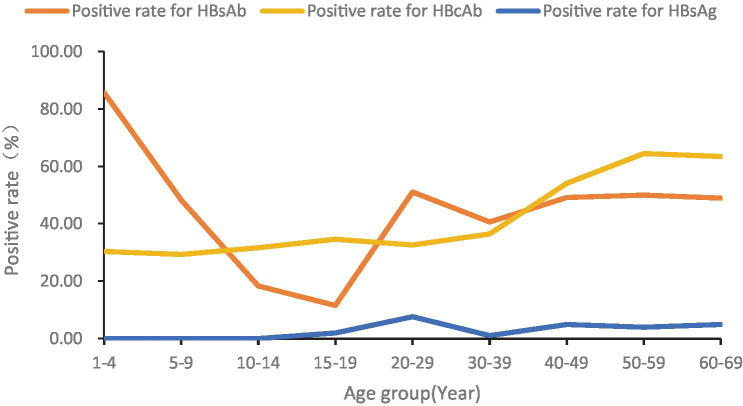
Positive rates of serum HBsAg, HBsAb and HBcAb in people aged 1–69.

Of all the 706 participants, 181 were aged 1–14 and 525 were 15–69 years old. There were 107 males and 74 females in 1–14 years old population. The serological test results for this population showed that there were no HBsAg positive, 171 HBsAg negative, and 10 with no results due to insufficient serum, 87 HBsAb positive, 87 HBsAb negative and 7 with no results due to insufficient serum, and 53 HBcAb positive, 121 HBcAb negative, and 7 with no results due to insufficient serum. Among the 15–69 years old population, there were 188 males and 337 females. The serological test results for this population showed that there were 22 HBsAg positive, 498 HBsAg negative, and 5 with no results due to insufficient serum; 231 HBsAb positive, 291 HBsAb negative, and 3 people with no results due to insufficient serum, and 257 HBcAb positive, 265 HBcAb negative, and 3 with no results due to insufficient serum. The overall HBsAg, HBsAb and HBcAb prevalence curves were seen in Figure 1.

### 3.2 Prevalence of HBsAb and HBcAb in participants aged 1–14

There were 181 people aged 1–14 years old in all, among which 7 people with no serum test results due to serum insufficiency were excluded from the analysis, and the remaining 174 people were included in the analysis. The overall positive rate of serum HBsAb was 50.00% (87/174), and the index increased from 1 year old, reached a peak of 90.91% at 2 years old, and then showed a significant downward trend (χ^2^ = 55.612, *p* < 0.001). The overall positive rate of HBcAb was 30.46% (53/174), which showed no obvious trend with age (χ^2^ = 0.065, *p* = 0.798).

#### 3.2.1 HBsAg prevalence in mothers of participants aged 1–14

Of all the mothers of 174 participants aged 1–14, there were 2 mothers recalled HBsAg positive and their children were 6 and 9 years old, respectively, when participated in our study. The 9-year-old child was HBcAb positive and another was negative. For the HBcAb positive child, both Hep B vaccination and HBIG were received within 24 h after born, while for another one, only Hep B vaccination was used. Among the rest, 107 mothers were HBsAg negative and 65 were failed to recall or had no idea of the test. Chi square test(χ^2^ = 0.68, *p* = 0.711) showed that there was no significant difference in children’s HBcAb positive rates among mothers with HBsAg positive, HBsAg negative and unknown HBsAg results.

#### 3.2.2 Hep B vaccination time of participants aged 1–14

All of the 174 participants have completed three-dose Hep B vaccination. From the perspective of vaccination time, there were 5 babies who did not get the first dose of Hep B vaccination within 24 h after born. There were 9 babies who got the second dose 30 days later than the appointed time(aged 1 month) and 20 babies got the last dose 30 days later than five months after the second dose. Chi square test(χ^2^ = 5.87, *p* = 0.015) showed that for the second dose, those who vaccinated 30 days later than the appointed time got higher HBcAb prevalence than those who vaccinated on time.

### 3.3 HBV prevalence in participants aged 15–69

There were 525 people aged 15–69 years old in total, among which 3 people with no serum test results due to serum insufficiency were excluded from the analysis. The remaining 522 people were included in the analysis. The overall positive rate of serum HBsAg, HBsAb and HBcAb in this study was 4.21% (22/522), 44.25% (231/522) and 49.23% (257/522), respectively. From the perspective of serological modes, among the HBsAg positive population, there were only 4 people with single HBsAb positive, 15 people with single HBcAb positive, 1 person with both HBsAb and HBcAb positive, and 2 people with both HBsAb and HBcAb negative. Whereas among the HBsAg negative population, there were 75 people with single HBsAb positive, 90 people with single HBcAb positive, 151 people with both HBsAb and HBcAb positive, and 184 people with both HBsAb and HBcAb negative.

#### 3.3.1 Analysis on influencing factors of HBsAb

Among 522 people, take HBsAb positive or not as dependent variable, and gender, age, occupation, education level, previous history of hepatitis, previous history of other liver diseases (such as fatty liver, alcoholic liver, etc.), and viral hepatitis vaccination status as independent variables in univariate analysis. The results showed that HBsAb positive was related to age, occupation, and previous diagnosis of other liver diseases (Table 1).

**Table 1:** Univariate analysis on influencing factors of HBsAb in people aged 15–69 in Huangpu District, Shanghai.

Variables		*N*	HBsAb(+)		HBsAb(−)		*χ* ^2^	*p*
			*N*	Proportion (%)	*N*	Proportion (%)		
Age							27.329	<0.001
	15–19	52	6	2.64	46	15.92		
	20–29	92	47	20.70	45	15.57		
	30–39	96	39	17.18	57	19.72		
	40–49	61	30	13.22	31	10.73		
	50–59	76	38	16.74	38	13.15		
	60–69	145	67	29.52	72	24.91		
Occupation							25.123	0.001
	Farmers	2	1	0.43	1	0.34		
	Worker	57	32	13.85	25	8.59		
	Officers and staff	71	35	15.15	36	12.37		
	Student	55	10	4.33	45	15.46		
	Teacher	10	3	1.30	7	2.41		
	Healthcare workers	24	6	2.60	18	6.19		
	Service personnel in public places	107	49	21.21	58	19.93		
	Retirement, unemployment, and others	196	95	41.13	101	34.71		
Whether other liver diseases have been previously diagnosed							5.078	0.038
	Yes	4	4	1.73	0	0		
	No	518	227	98.27	291	100		

All the variables with statistical significance (*p* < 0.05) in univariate analysis were included in the multivariate logistic regression model. Age 15–19, occupation as farmers, not previously diagnosed with other liver diseases were taken as reference during multivariate analysis and the entering level was specified to be 0.05 and the exclusion level to be 0.10. The results showed that age was the only influencing factor of HBsAb positive. The OR values for the age group 20–29, 30–39, 40–49, 50–59 and 60–69 were 8.007(*95%CI for OR*: 3.116–20.578), 5.246(*95%CI for OR:* 2.043–13.472), 7.419(*95%CI for OR:* 2.763–19.923), 7.263(*95%CI for OR:* 2.767–19.067) and 6.921(*95%CI for OR:* 2.774–17.271), respectively.

#### 3.3.2 Additional examinations for HBsAg positive participants

There were 22 HBsAg positive participants aged 15–69 included in our additional examinations including ALT, AST, total bilirubin, albumin, globulin, liver fibroscan and liver ultrasound. Of all the 22 participants, 10 refused to follow the examination and the left 12 completed all the examination. One participant got higher indexes of ALT, AST and liver fibroscan and evaluated as liver inflammatory lesions by liver ultrasound without antivirus treatment. The rest 11 were tested within the normal range of all the indexes except that liver ultrasound showed that 7 were normal liver and 4 were liver inflammatory lesions. Only 16.67%(2/12) of the participants received antivirus treatment. Of all the participants, 33.33%(4/12) had relatives with HBV infection, among which 3 were siblings and 1 was father.

#### 3.3.3 Analysis on influencing factors of HBcAb

Among 522 people, univariate analysis was carried out with HBcAb positive or not as dependent variable and with gender, age, occupation, education level, previous history of hepatitis, previous history of other liver diseases (such as fatty liver, alcoholic liver, etc.) and viral hepatitis vaccination status as independent variables. The results showed that HBcAb positive was related to age, education level, occupation, previous diagnosis of fatty liver, previous HepB vaccination, and HBsAb positive (Table 2).

**Table 2:** Univariate analysis on influencing factors of HBcAb in people aged 15–69 in Huangpu District, Shanghai.

Variables		*N*	HbcAb(+)		HBcAb(−)		*χ* ^2^	*p*
			*N*	Proportion (%)	*N*	Proportion (%)		
Age							39.543	<0.001
	15–19	52	18	7.11	34	12.93		
	20–29	92	30	11.86	62	23.57		
	30–39	96	35	13.83	61	23.19		
	40–49	61	33	13.04	28	10.65		
	50–59	76	49	19.37	27	10.27		
	60–69	139	88	34.78	51	19.39		
Education level								
	>Senior school	202	122	47.47	80	30.19	16.426	<0.001
	≤Senior school	320	135	52.53	185	69.81		
Occupation							19.675	0.006
	Farmers	2	1	0.39	1	0.38		
	Worker	57	33	12.84	24	9.06		
	Officers and staff	71	26	10.12	45	16.98		
	Student	55	21	8.17	34	12.83		
	Teacher	10	5	1.95	5	1.89		
	Healthcare workers	24	9	3.50	15	5.66		
	Service personnel in public places	107	46	17.90	61	23.02		
	Retirement, unemployment, and others	196	116	45.14	80	30.19		
Whether fatty liver has been previously diagnosed							5.794	0.016
	Yes	85	52	20.23	33	12.45		
	No	437	205	79.77	232	87.55		
Previous hepatitis B vaccination							12.926	0.002
	Yes	169	64	24.90	105	39.62		
	No	246	135	52.53	111	41.89		
	Unknown	107	58	22.57	49	18.49		
HBsAb test results							45.504	<0.001
	Positive	231	152	59.14	79	29.81		
	Negative	291	105	40.86	186	70.19		

All the variables with statistical significance (*p* < 0.05) in univariate analysis were included in the multivariate logistic regression model. Age 15–19, education level ≤ senior school, occupation as farmers, not previously diagnosed with fatty liver, unknown of previous Hep B vaccination and HBsAb negative were taken as reference during multivariate analysis. The entering level and exclusion level were specified to be 0.05 and 0.10, respectively. The results showed that elder age, lower education level, and HBsAb positive were the influencing factors of HBcAb positive (Table 3).

**Table 3:** Multivariate logistic regression analysis on influencing factors of HBcAb in people aged 15–69 in Huangpu District, Shanghai.

Variables	*β*	*Wald* χ^2^	*p*	*OR*	95%*CI for OR*
Age(15–19 as a reference)					
50–59	0.766	3.771	0.052	2.152	0.993-4.663
60–69	0.814	5.259	0.022	2.258	1.126-4.529
Education level	−0.487	4.958	0.026	0.615	0.401-0.943
HBsAb	1.25	38.672	<0.001	3.490	2.354-5.174

## 4 Discussion

In 1992, China officially announced to promote HepB vaccination on a nationwide scale to reduce HBV infection (11) after trials within some provinces, including Huangpu district of Shanghai, for six years. Afterwards, HepB vaccine was included into the National Immunization Program(NIP) in 2002, namely the strategy of neonatal HepB vaccination has been implemented since then, which was also a globally recommended strategy for intermediate- and high-endemic regions (12). The 2014 national seroepidemiological survey of hepatitis B infection showed that (13) the prevalence of HBsAg in people aged 1–14 years was less than 1%, and the prevalence of HBsAg in people aged 1–14 years in this study was 0.00%, which better maintained the WHO hepatitis B prevention and control target in the western Pacific region. The prevalence of HBsAg in people aged 15–69 years was 4.21%. Considering that age-specific increasing HBsAg prevalence was more likely related to the carry-over of the infection occurred in early life (14), mostly due to imperfect accessibility or coverage of HepB vaccination at the beginning of its introduction, it was commonly recognized in the real world that HepB vaccination had played a very important role in the process of HBV infection control and prevention (15).

HBsAb is a protective antibody against HBV infection in human bodies, and most newborns can produce protective antibodies after receiving HepB vaccination. However, the HBsAb levels will continue to decline with age, and even cannot be detected in serum samples after decades. In this study, the HBsAb positive rate in people aged 1–14 years also decreased gradually with age, and the HBsAb positive rate in 14-year-old people was 0. Some people may not respond after HepB vaccination, and some people may find it in themselves that serum HBsAb was below the detection limit about 10 years after HepB vaccination (16). Although serum HBsAb cannot be detected, studies have shown (17) that cellular immunity to HBV existed in human bodies after HepB vaccination, which could still clear HBV from the body to a certain extent. As a result, the current HepB vaccine booster strategy in general population still needs to be discussed (18, 19), except for some special occupational population such as surgeons and emergency physicians (20). It is also recommended to regularly monitor their serum HBsAb levels due to their relatively more frequent exposure in daily operations and if the test result is below the detection limit, HepB vaccine booster would be strongly recommended to prevent possible occupational HBV infection.

The HBcAb in children is largely derived from mother-borne antibodies (21). In our study, it was very pitiful that only HBsAg results of the mothers were recorded. Apart from two mothers with HBsAg positive, we still had more than 30 % of the mothers not knowing the HBsAg status themselves, which made it more hard to tell if it’s the mother-to-child way to get the offspring infected. The positive rate of HBcAb in people aged 1–14 years in this study reached 30.46%, which was pretty higher than the survey results in Qinghai and other places in the same period (22, 23). Considering that all respondents aged 1–14 years have accepted three-dose HepB vaccination according to the questionnaire, and studies showed that latent HBV infection in HBcAb positive individuals with successful vaccination is extremely rare (24, 25), the factors affecting the HBcAb positive rate might be related to possible non-timely vaccination in early life (26) or close contact infection within the family. Although the three-dose Hep B vaccination of individuals aged 1–14 was relatively perfect, lack of the timeliness of the second dose was responsible for HBcAb positive to some extent in this study. Few studies (27) have reported recently that lack of timeliness of the birth dose are likely to have contributed to HBV infection in vaccinated population. There were fewer studies reporting the relation between the second dose and HBV infection. As a result, it is still necessary to evaluate the timeliness of vaccination when the coverage is good and close to 99.9% in China (28). Furthermore, the neighborhood committees selected in this survey are in the process of demolition according to the urban planning, so the composition of the permanent population is relatively complex. For example, most of the population belonged to immigrants from other provinces with low incomes and poor hygiene concept and living habits which made health interventions, such as using separate toothbrushes, more difficult to do. In addition, the parents’ HBV infection status was not clear, thus resulting in HBV infection through close contact within the family (29). Despite of the high HBcAb positive rate, most of the individuals would be self-resolved (19), but a recent published study showed (30) that children who had history of hospital admission were more likely to be HBcAb positive. As a result, the reason for HBcAb positive is still worth being studied.

The results of the study on people aged 15–69 years showed that age was the influencing factor of serum HBsAb positive rate. On one hand, serum HBsAb levels will decrease with age (31). On the other hand, those who were tested HBsAb positive in this study were mostly accompanied with HBcAb positive which indicated the possibility of previous infection. Considering that HepB vaccine was included in immunization plan in 1992, people aged 28 and above in this study may not get timely HepB vaccination at birth or after, so in most situation they naturally acquired HBV immunity from infection through close contact within the family or medical operations in hospitals.

According to the serological modes of HBV infection in people aged 15–69, more than 70% people were also detected HBcAb positive within HBsAg positive population in this study, which indicated a status of chronic HBV infection. Whatever he who had accepted HepB vaccine or not, HBcAb positive was an independent prognostic factor for HBV-related HCC patients (32), which showed that health education on early antiviral therapies to HBV infection population was of great importance in case they would progress into cirrhosis or liver cancer.

The positive rate of HBcAb in the age group over 50 years old was higher than that in <20 age group. The possible reason for the result might be the poor coverage of Hep B vaccination fifty years ago and HBV infection was commonly acquired through close contact in family members. In addition, China’s sanitary conditions or principles varied according to hospitals and were relatively far from satisfaction in last century, so a large amount of HBV infection was caused by irregular medical operations. General population, even healthcare workers themselves had no idea of HBV infection (33), which emphasized the importance to regulate the medical operations and raise the awareness against HBV infection to reduce hospital infection as well as protect healthcare workers. Another finding of the study was that high education level was considered a protective factor for HBV infection, probably because people who were well educated would accept knowledge on disease prevention more objective. Recent studies (34, 35) showed that the public awareness of HBV infection was insufficient and there was still social discrimination against HBV infection in adults, and explained that people with high education level may be more receptive to relevant disease prevention knowledge, more personal health awareness, more attention to personal hygiene and healthy living habits, which could better help prevent HBV infection.

There were still shortcomings in this study. First of all, the reason for the high HBcAb positive rate in people aged 1–14 years was still uncertain, and the study on HBV infection status of family members of those HBcAb positive children was planned to carry out afterwards in order to clarify the possibility of close contact infection within the family. Secondly, the results of this study showed that the HBcAb positive rate in the population was high overall, which to some extent supported that it was necessary to regularly investigate the HBV infection status in people of different ages so as to update the local risk factors of HBV infection and provide guidance for the key points of following work.

In summary, the immunization strategy of HepB vaccine in newborns has contributed a lot in reducing HBsAg prevalence. However, the overall HBcAb positive rate is still high in Huangpu district, Shanghai, for which the reasons including the timeliness of vaccination and tranmission within families or through medical injuries are of great importance to be further explored. Therefore, it is still recommended that preventive measures should be taken in the following aspects: one is to continue applying the HepB vaccination policy to newborns and to carry out re-immunization or booster immunization for non-responders or high-risk groups according to individual circumstances; The second is to regularly monitor HBV infection in the population of all ages to provide a basis for HBV prevention and control; The third is to strengthen health education on cultivating correct hygiene habits against HBV infection within family members, especially for key population groups such as HBV carriers and immigrants, so as to further reduce HBV infection. Last but not the least, efforts should be put on eliminate social discrimination against HBV infection, encouraging HBV infected individuals to get diagnosed and accept antiviral therapy as early as possible in order to reduce disease burden like HCC in the future.

## Data availability statement

The raw data supporting the conclusions of this article will be made available by the authors, without undue reservation.

## Ethics statement

The studies involving humans were approved by Ethics Committee of the Chinese National Center for Disease Control and Prevention (No.201811). The studies were conducted in accordance with the local legislation and institutional requirements. Written informed consent for participation in this study was provided by the participants' legal guardians/next of kin. Written informed consent was obtained from the individual(s), and minor(s)' legal guardian/next of kin, for the publication of any potentially identifiable images or data included in this article.

## Author contributions

WY: Data curation, Formal Analysis, Writing – original draft, Conceptualization, Investigation. SM: Data curation, Investigation, Writing – original draft. CJ: Data curation, Investigation, Writing – original draft. SF: Formal analysis, Methodology, Supervision, Writing – original draft. RH: Project administration, Supervision, Writing – review & editing. YY: Funding acquisition, Writing – review & editing, Software.
